# Exploring the perspectives of urban and regional living Aboriginal and Torres Strait Islander Peoples regarding bush foods, nutrition and health: insights for culturally informed health policy in Australia

**DOI:** 10.1017/S1368980025100694

**Published:** 2025-07-17

**Authors:** Jessica Cartwright, Niall Turner, Sherie Bruce, Yasmina F. Sultanbawa, Michael E. Netzel, Olivia R.L. Wright

**Affiliations:** 1 School of Human Movement and Nutrition Sciences, The University of Queensland, Brisbane, QLD, Australia; 2 ARC Industrial Transformation Training Centre for Uniquely Australian Foods, Queensland Alliance for Agriculture and Food Innovation (QAAFI), The University of Queensland, Brisbane, QLD, Australia

**Keywords:** Public health, First Nations, Bush foods, Nutrition, Children, Urban, Regional, Aboriginal health

## Abstract

**Objective:**

This study aims to explore the perspectives of urban and regional living Aboriginal and Torres Strait Islander adults and children regarding Bush Foods, nutrition and health to advocate for future culturally informed programmes and policy.

**Design:**

The qualitative study conducted nine Yarning sessions, which were recorded and transcribed verbatim. An inductive, reflexive thematic analysis using a codebook was employed to analyse the data.

**Setting:**

All Yarns were conducted face-to-face in various locations across Southeast Queensland.

**Participants:**

Yarning sessions were conducted with Aboriginal and Torres Strait Islander participants (*n* 20), including ten adults and ten children. Participants resided in areas classified as inner regional, outer regional and major cities.

**Results:**

Five interconnected themes were generated concerning participants’ perspectives on Bush Foods, nutrition and health. These themes included the effects of colonisation and bureaucratic impositions, socio-environmental factors influencing food provision, the significance of Bush Foods in cultural connection and nutritional health, the importance of reciprocity in communities and the nuanced role of agency influenced by education.

**Conclusions:**

The findings were synthesised into two overarching concepts: the role of family, kin and culture at the individual and community level, aligning with cultural determinants of Indigenous health, and the broader socio-political influences of colonialism, capitalism and power imbalances, reflecting social determinants of Indigenous health. This research highlights a need for culturally informed health policies guided by consideration of cultural, social and commercial determinants that support an Indigenised food system and Bush Food reintegration for urban-living Aboriginal and Torres Strait Islander adults and children.

For over 60 000 years prior to the British invasion and colonisation of Australia, the food systems of Aboriginal and Torres Strait Islander Peoples were complex, varied and highly sophisticated^(1,2)^. Provisions of food were borne of Aboriginal and Torres Strait Islander ways of knowing, being and doing – complex scientific knowledge and land care practices that centred around reciprocity with Country and community^(1,2)^. Traditional diets were characterised by their exceptional nutritional content, devoid of refined sugars and abundant in nutrients, protein, complex carbohydrates and unsaturated fats^(1,2)^. Following colonisation, Aboriginal and Torres Strait Islander Peoples were forcibly disconnected from their traditional food systems^(1,2)^. The implementation of violent colonial practices, including the forced removal from ancestral homelands, assimilation policies and food rationing, catalysed a profound shift in dietary patterns and lifestyle choices^(1,2)^. The transformation of the food landscape and government-imposed restrictions on food acquisition resulted in a modern reliance on western foods and food systems, a phenomenon referred to as ‘nutritional colonisation’^(1)^. This paradigm, compounded by systemic racism and inadequate policy frameworks, has contributed to an increased incidence of non-communicable diseases, heightened levels of food insecurity and a pronounced health disparity between Aboriginal and Torres Strait Islander Peoples and non-Indigenous Australians^(1,2)^. The socio-political and socio-economic context of a population is known to impact living and working conditions, behaviours and social and emotional wellbeing, all of which ultimately affect equity in health^(3)^. However, despite ongoing colonial practices and a western-dominated food system, many Aboriginal and Torres Strait Islander Peoples continue holding ancestral knowledge and utilise traditional foods to support health and wellbeing^(4)^. This is an example of how the cultural determinants of health, ‘family/community, Country and place, cultural identity and self-determination’ can impact health and wellbeing outcomes for First Nations People^(5)^.

Rectifying the inequities facing Indigenous Peoples has been identified as a priority in both national and global health policies. The 2007 United Nations Declaration on the Rights of Indigenous Peoples (UNDRIP) outlined specific rights for Indigenous populations worldwide, including the right to self-determination and the support of Traditional Knowledge systems^(6)^. Australia, a signatory of UNDRIP, has faced criticism for its inadequate response to these commitments^(7)^. In Australia, successive governments have introduced national policies aimed at improving the health outcomes for Aboriginal and Torres Strait Islander Peoples, including the National Agreement on Closing the Gap^(8)^ and the National Aboriginal and Torres Strait Islander Health Plan^(9)^ (NATSIHP) 2021–2031. However, policies have historically had mixed success and have been consistently criticised for their lack of tangible outcomes for Aboriginal and Torres Strait Islander Peoples^(10)^. The shortcomings of previous policies, along with various nutritional and public health programmes, have been attributed to a deficit-based approach, lack of resources, political will and implementation plans and the failure to consider the perspectives and priorities of diverse Aboriginal and Torres Strait Islander communities^(11,12)^. Therefore, it is crucial to prioritise the voices and lived experiences of Aboriginal and Torres Strait Islander Peoples in addressing health and nutrition disparities and in the development of future policies.

There is considerable literature documenting the importance of connection to culture and Traditional Foods for the health of Aboriginal and Torres Strait Islander Peoples, with a focus on communities living in rural and remote areas^(13,14)^. For example, Cubillo *et al.* (2023) recently investigated nutrition, health and traditional foods in the context of a remote Aboriginal community^(15)^. While this research can, and does, help inform policy, the lack of literature from major cities and regional areas is concerning, as this is where the majority of Aboriginal and Torres Strait Islander Peoples reside^(14)^. For example, a strategy for remote Indigenous food security within Australia is currently under development, yet there is no equivalent policy for urban Aboriginal and Torres Strait Islander communities^(16)^. The relative lack of research and policy interest in these urban populations may stem from a perception that research should prioritise remote communities due to the disproportionate burden of illness experienced from factors such as limited access to health services^(17)^. However, other research suggests that Aboriginal and Torres Strait Islander Peoples living in urban and regional areas experience health disparities comparable to those faced by their remote counterparts and in fact encounter additional layers of marginalisation, minority status and invisibility^(13,14)^. This highlights a significant gap in the research that necessitates further exploration – it is important to listen to the voices of Indigenous People living in more urban areas and similarly harness their views and experiences to inform policy^(14)^.

One qualitative study worth noting that has been conducted by McCarthy *et al.* (2018) with Aboriginal and Torres Strait Islander families living in urban areas is around food security experiences^(18)^; however, it was centred around only adult perspectives. Health and nutrition research with Aboriginal and Torres Strait Islander communities is largely conducted with adult participants^(2,19,20)^. Considering that over 30 % of Aboriginal and Torres Strait Islander Peoples are under the age of 15^(21)^ and that these children are future knowledge holders, representing cultural continuity and research into the priorities, perspectives and insights of this group is critical in the development of equitable policies and programmes^(19,20)^. The lack of representation has been noted by several studies, which have highlighted the need for further exploration into health and wellbeing perspectives across the lifespan, including those of children^(2)^.

The role of culture and traditional Bush Foods in the health of Aboriginal and Torres Strait Islander Peoples, especially among younger populations living in urban and regional areas, remains underexplored. Prioritising these perspectives is crucial for a comprehensive understanding of health and nutrition within these communities, which can act to shape culturally informed policy. However, strong connections between culture and social and emotional wellbeing are being established^(22)^. It is positive to note this research area is gaining much-needed attention, with a recent publication by Anderson *et al.* (2024) establishing important connections between food and wellbeing for First Nations People^(4)^ around Australia, creating a strong foundation for this current study to build off and compare against in an urban-specific context.

Therefore, the present study aims to explore the experiences, perspectives and insights of urban and regional living Aboriginal and Torres Strait Islander adults and children regarding Bush Foods, nutrition and health. Importantly, this research gap is of particular interest to the Indigenous partners that are affiliated with our research centre, whose aim is to improve the health of their communities through Bush Foods. Ultimately, this will amplify the voices of urban and regional living Aboriginal and Torres Strait Islander Peoples, which may inform future transformative policy and programmes to promote health equity in Australia.

## Methods

### Indigenous governance

The project approach was designed and reviewed by the First Nations Advisory Group and the First Nations Enterprise Group at the Australian Research Council Industrial Transformation Training Centre for Uniquely Australian Foods (UAF) at the University of Queensland (UQ), Australia, as per the outlined governance structure of the research centre^(23)^. The chair of the First Nations Enterprise Group provided additional insights and advice relating to data collection and data analysis. T.B., a Bundjalung man and Indigenous academic, offered important cultural considerations related to Yarning in the conceptualisation of this project. K.W., a proud Ngarabal person with vast experience working in qualitative health research, provided guidance on appropriate methodology and analysis to ensure cultural competence and responsiveness. Furthermore, S.B., a proud Arrernte and Yolngu woman and emerging researcher, was involved heavily in every phase of the project from conceptualisation and ethics application to data collection and analysis. Although this research project was not carried out by an entirely Indigenous-led research team, all key decisions related to this research were guided and informed by Indigenous perspectives.

Strategies to ensure cultural rigour involved having robust discussions with various Indigenous People, both within and outside the authorship team, within and outside UAF and within and outside of academia, throughout the research process. We also ensured alignment with NHMRC^(24)^ and AIATSIS^(25)^ guidelines for ethical conduct in research with Aboriginal and Torres Strait Islander Peoples. We used the CONSIDER statement^(26)^ to strengthen reporting of health research involving Indigenous Peoples in conjunction with the CREATE tool^(27)^, which is used to assess the quality of Indigenous health research.

### Positionality of the research team

Our research team appreciates the importance of reflexivity in qualitative research, acknowledging how one’s own worldviews, assumptions and lived experience may influence results. All authors on this paper have experience with Indigenous health research, a passion for working with Aboriginal and Torres Strait Islander Peoples and communities, and have diverse skillsets and research backgrounds: J.C., O.W. and N.T. are dietitians with community health experience, and S.B., M.N. and Y.S. are food/nutrition scientists. Please see Supplementary Material (SM) 1 for further information on the positionality of the authors.

While the non-Indigenous authors cannot ever truly understand the lived experience of being an Indigenous Person in this world, we empathise with the journey towards self-determination and wish our research to align with, and assist in, this journey. We also collectively acknowledge the history of traditional, repressive research practices that have reinforced western ontologies and epistemologies to the detriment of Indigenous worldviews^(28)^.

All authors engaged in deep reflexivity, critically examining our worldviews, which we unanimously found to align with an Indigenous axiology, particularly the concept of relational accountability and reciprocity^(29)^, meaning we strive to undo imbalances of power and contribute to change in the deficit framing of Aboriginal and Torres Strait Islander Peoples’ health discourse^(28)^. Therefore, we employed a decentring approach in this research, which involves understanding and undoing privilege through learning from the other rather than learning about the other^(30)^. However, to ensure high-quality research, given that the majority of the authors are non-Indigenous researchers working within this space and that our research aims to privilege the voices of First Nations participants, it was most pragmatic to adopt a critical realist ontological lens^(31–33)^. Please see SM1 for further details on why critical realism was important to allow privileging of participant voices while ensuring methodological and cultural rigour.

### Participant recruitment

Aboriginal and Torres Strait Islander adults and children were purposively recruited between August 2023 and February 2024 via group email (*n* 7) and in-person/individual correspondence (*n* 13), primarily through previously established connections with the research team and UAF. We incorporated snowball sampling from existing networks to access a wider range of participants.

Eligibility criteria for participation in this study were as follows: (i) identify as an Aboriginal and/or Torres Strait Islander person, (ii) be >7 years old, (iii) be able to speak and understand the English language, (iv) have signed consent (from parents/guardians where necessary), (v) reside in or visit South-East Queensland during the data collection period and (vi) live in an area classified as inner regional, outer regional or a major city, according to the Australian Statistical Geography Standard-Remoteness Area tool^(34)^, which is commonly used in health research in Australia. Participants excluded from the study were those currently living in areas classified as remote or very remote.

All participants received a small gift for their contribution to the study. Other additional non-monetary benefits were discussed in advance so that participants knew what they would receive for their engagement.

### Data collection

Data were collected through nine in-person, audio-recorded Yarning circles. The practice of Yarning has been a keystone of Aboriginal and Torres Strait Islander cultures for millennia^(35)^. Grounded in elements such as relationality, storytelling and flexibility, it ensures participants have autonomy to drive the conversation in a direction that feels most appropriate for them^(35)^. It has been widely recognised as a valuable method for exploring qualitative questions in research with Aboriginal and Torres Strait Islander Peoples as it builds trust and fosters culturally safe engagement^(35–37)^.

Yarns were conducted with groups of one to five Aboriginal and Torres Strait Islander adults or children and ranged in duration from 20 min to 1 h. All conversations began with a social Yarn, which moved into research topic Yarning. Unlike formal interviewing, research Yarning does not adhere to rigid structures^(35,36)^. Instead, researchers encourage participants to share their stories from the vantage point of lived experience, allowing for topics, opinions and experiences to emerge organically^(35,36)^. The Yarns were facilitated by non-Indigenous researcher J.C. and Aboriginal researcher S.B. when available and/or when additional cultural insight or connection was required (e.g. when J.C. had not met the participants previously)^(38)^. Three broad topics were raised by the facilitator(s) in each Yarn – (1) popular/favourite food and drinks and why, (2) familiarity with Bush Foods and thoughts/knowledge related to them and (3) perceived importance of nutrition and health and if/how this is considered when purchasing or consuming food and drinks.

The use of open-ended questions combined with a flexible questioning style allowed for open reflection and discussion of participants’ perspectives, insights and knowledge relating to the research question. Group dynamics were managed by ensuring all participants in a Yarning circle had prior connections to all other participants they were Yarning with, fostering a safe and comfortable environment for open sharing. Occasionally (more so with younger participants), strategies such as going around the circle to offer thoughts on a topic or question were employed to ensure the voices of all participants were heard. In the Yarns with children, further inquiries and follow-up questions were also used when needed. As required, the facilitator(s) incorporated therapeutic Yarning to ensure an affirming and non-judgemental space was created^(35)^.

### Data management

The audio recordings of the Yarning sessions were transcribed verbatim by J.C. Throughout the transcription process and subsequent data reporting, each participant was allocated a pseudonym comprising their Yarn number and participant number within the Yarn. This practice was adopted to maintain the confidentiality of all participants involved in the study. All documents relevant to this study were stored in UQ Research Data Manager, a password-protected online filing system that only the authors can access.

### Data analyses

Analysing the Yarns within a critical realist ontology was pragmatically necessary in this project as it aligned with our commitment to obtain high-quality insights while centring the perspectives of Indigenous participants^(31–33,39)^. Recognising the influence our roles as researchers can have on the analytical process, we were particularly mindful of ensuring that the voices of Aboriginal and Torres Strait Islander participants are central to shaping the research outcomes. Therefore, this study sits most appropriately within what Braun and Clarke (2022) have coined to be a ‘Medium Q’ qualitative thematic analysis orientation^(39)^. For the same reasons, the researchers used semantic and experiential coding, drawing on the perspectives, knowledge, insights and lived experience of the participants.

Researchers initially familiarised themselves with all transcripts, with the four lead authors (J.C., N.T., S.B., O.W.) independently generating initial codes inductively and then collaboratively searching for themes representative of these codes. Draft themes and codes were defined by these four authors to establish a codebook^(40)^. Three researchers (J.C., N.T., S.B.) were then assigned 4–5 transcripts each to import into NVivo12 software (QSR International) and apply the codebook, continuously discussing and cross-checking coding throughout the process. The codebook was iteratively updated by J.C, S.B. and N.T. as further meaning was generated from the transcripts, recoding each time as necessary. Refer to SM2 to view the codebook used by the researchers. This approach allowed the researchers to remain reflexive in the analysis while also ensuring cultural competency and responsiveness through collaborative coding, intercoder agreement and participant checking^(39)^. All researchers kept a journal of reflections throughout the research process and consistently discussed these reflections with the research team. Refer to SM1 for further details of this hybrid approach to data analysis and explanation of why this was necessary to ensure theoretical and methodological coherence.

## Results

### Participant demographics

The Yarns engaged a total of twenty Aboriginal and Torres Strait Islander participants (0 % drop-out rate), whose demographic characteristics are presented in Table 1. The age of participants ranged from 11 to 70 years old. The largest proportion of participants resided in areas classified as inner regional, with thirteen participants living in Queensland, two participants living in New South Wales and five living in the Northern Territory.

**Table 1.** Key participant demographic information

Participant characteristics	RA1 (*n* 5)	RA2 (*n* 9)	RA3 (*n* 6)	Total	% of participants
Female	5	4	2	11	55 %
Male	0	5	4	9	45 %
Child (<18 yo)	3	7	0	10	50 %
Adult (>18 yo)	2	2	6	10	50 %

Yo, years old; RA, Remoteness Area according to Australian Statistical Geography Standards^(34)^ where RA1 = major city, RA2 = inner regional, RA3 = outer regional.

### Qualitative results

**Figure 1.**
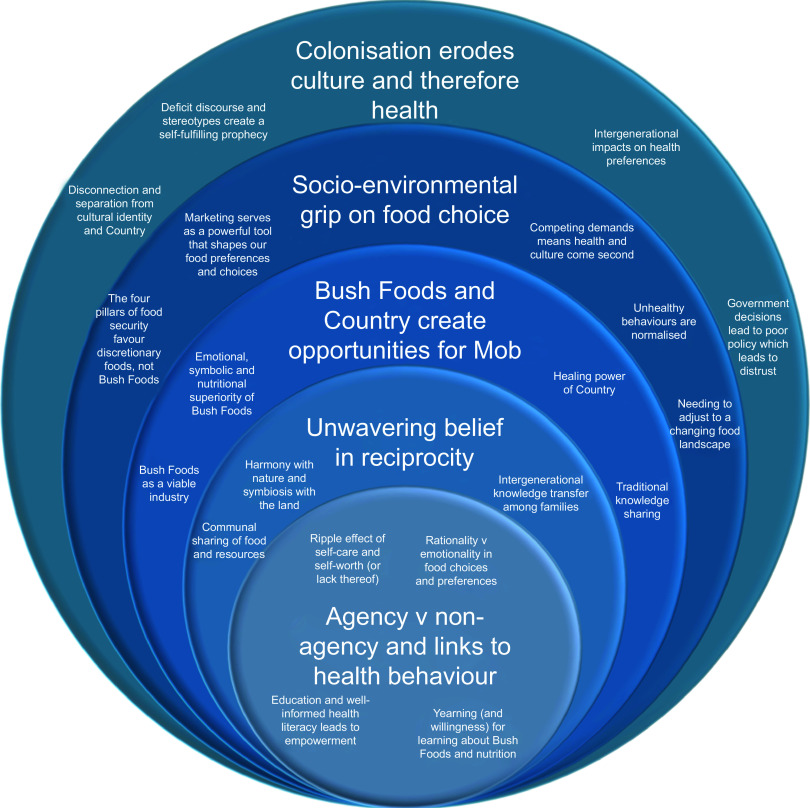
Insight circle representing the main themes and codes generated from analysis of Yarning circle data, highlighting how the outer circles influence the inner circles.

The qualitative analysis generated five central themes from the Yarns revolving around the ramifications of colonisation and socio-environmental factors; the significance of Bush Foods, Country and culture; the enduring reciprocity within communities and for Country; and the nuanced concept of agency (Fig. 1). These themes were intricately interconnected, rooted in both negative experiences linked to colonisation and capitalism and positive perceptions stemming from family and culture.

#### Colonisation erodes culture and therefore health

This was the broadest theme, being one that was perceived to drastically impact culture, nutrition and health both historically and contemporarily. Participants recounted experiences of being subjected to policies and bureaucratic structures that dictated their way of life, including their diet, living conditions and connection to culture. A prevailing sentiment of distrust towards the government and westernised food systems emerged, with adult participants in particular highlighting the prioritisation of market interests and capital over the wellbeing of Aboriginal and Torres Strait Islander communities. In particular, the ongoing lack of community consultation was identified as a significant issue perpetuating the cycle of ineffective policy decisions driven by governmental intervention. This absence of meaningful consultation extended to the provision of tailored services in communities and the inadequate representation of Aboriginal and Torres Strait Islander Peoples in positions of power. The resulting lack of representation contributed to a broader sense of distrust in authority and underscored the failure of previous policies to address the unique challenges faced by Aboriginal and Torres Strait Islander communities.

‘It’s not about the community. When it comes to the land, you got the same problem – the wrong people in those roles…And then the people say, “well, why is all this not working?” You know what I mean… And then you go in every community and people that run everything are non-Indigenous.’ *(P5.5, male adult, outer regional)*

Participants reflected on the lasting consequences of policies like ‘The Intervention’^(41)^ and the ‘Services Australia Basicscard’^(41)^ on their health behaviours, choices and overall wellbeing. These policies were seen not only as infringements on individual rights and autonomy but also as ineffective measures that contributed to increased prices of goods and services in communities. While some positive aspects, such as healthy eating programmes and increased protection for women, were acknowledged, participants criticised the top-down approach and perceived the ‘governments telling [them] what to do’ *(P4·2, female adult, major city)* imposed on community decision-making processes. Additionally, bureaucratic hurdles, referred to as ‘red tape’ *(P1·3, male adult, outer regional)*, were cited as barriers to self-determination. Particularly, government policies requiring permits or leases for harvesting traditional foods from Country were seen as ‘not recognising Native Title rights’ *(P1·1, male adult, outer regional)*, impacting community economics and health. While discussions around bureaucracy and policy were generally discussed pessimistically by adults, it was interesting to note that the same pessimism towards the government was not conveyed by children. Specifically, it was not brought up at all by younger child participants, yet it was acknowledged and deemed a relevant discussion point by older child participants. Comments by older children such as ‘[ancestors] ate [traditional foods] before, then it was taken away’ *(P6·1, female child, inner regional)* and ‘I’ve seen, like, the Stolen Generation, a lot of people can’t really like…. get in touch with that stuff anymore because it’s just been lost’ *(P7·1, female child, major city)* highlight this.

Several participants recounted narratives of forced removal and familial separation from their ancestral lands due to the policies of the Stolen Generation and mission systems. This displacement resulted in a profound disconnection from cultural identity and Country, exerting a significant influence on participants’ nutrition and health. Adult participants shared their ongoing struggle to trace their lineage due to historical disruptions inflicted upon their ancestors and highlighted the western lack of understanding regarding the intrinsic significance of place and Country for Aboriginal and Torres Strait Islander Peoples’ wellbeing. One participant vividly expressed the challenges of navigating familial trauma stemming from removal, recounting the stigma associated with their identity.

‘I didn’t grow up in a household where my culture was strong… my grandmother was part of the Stolen Generation… so it was almost taboo to kinda bring it up… Now I have an appreciation why it was like that. Like I always felt like it was kind of held back from me… it wasn’t held back from me because it wasn’t available! So, for me, in the line of work I’ve chosen to take, I’ve actually taught a lot of my culture back to my mum and dad about how we work.’ *(P1.1, male adult, outer regional)*

The profound impacts of cultural disconnection were also a significant concern among child participants, who expressed feelings of psychological distress due to their perceived lack of connection to their culture. As one participant lamented, ‘I don’t feel like I’m that in deep with it, which is sad’ *(P6·1, female child, inner regional)*, highlighting the emotional toll of this disconnection. This sentiment was echoed by other young participants, emphasising that the challenges of reconnecting with their culture when familial ties are strained or when ancestral heritage is unclear remained a poignant reality.

Disconnection from traditional lands was further compounded by experiences of racism, both overt and systemic. Participants shared difficulties in overcoming negative stereotypes and biases, which perpetuated deficit discourse and contributed to feelings of low self-confidence and diminished expectations.

‘Well, he’s 16 now. He can’t go to school straight away cause of the colour of his skin. They go “yeah no get out you little shit”… and so he walks off and he’s got the cops on his back.’ *(P5.1, male adult, inner regional)*

The ongoing intergenerational trauma of forced removal, mission systems and other racist policies was also seen by participants to directly affect their food choices and other health behaviours. Participants often described dependence on high-calorie, low-nutrition foods as an ‘addiction’ that is an intergenerational vestige of living on missions. Referring to the pervasive presence of diabetes in the community, a participant recounted, ‘Everyone that’s come off the mission… they’ve already got [diabetes]… The ration system that was fed to them was the sugar, flour, tobacco, tea… Every generation’s had issues with that…’ *(P1·3, male adult, outer regional)*

#### The socio-environmental grip on food choice

Participants emphasised the profound influence of social and environmental factors on their food choices and health behaviours. Food security and insecurity emerged as a central focus, with participants highlighting the four pillars of food security – availability, access, utilisation and stability, as critical determinants of their dietary decisions. The convenience of easily available high-calorie or fast foods also often outweighed considerations of health and nutrition. Many participants described how limited access to affordable, nutritious options often compelled them to opt for inexpensive, low-nutrition foods, despite recognising the health implications of such choices.

‘Having food in the house, regardless of its nutritional quality, is probably the first thing. If you had food – you’re probably at more of an advantage than most people growing up. Yeah. So regardless of the nutritional quality of that food… at least you’d be in bed not starving.’ *(P1.1, male adult, outer regional)*

Participants similarly described that competing demands often resulted in healthy choices and cultural practices being relegated to a secondary position, unless it was made an explicit priority. Responsibilities with work, financial stability and general busy schedules meant that convenience foods were chosen by adult participants ‘because of time constraints’ *(P1·2, male adult, outer regional).* Meanwhile, children discussed being at the whim of what their parents decide.

‘At the moment our dad’s like, he works a lot of night shifts because he’s in the ambulance and our mums in her like reporting phase because she’s a teacher. So she’s doing a lot of reports and it’s… Just easier to just like buy something that’s ready.’ *(P6.2, male child, inner regional)*

Participants expressed that residing in a capitalist environment limited their engagement with culture. However, they noted this quality of culture taking second place is less evident when they visit remote communities where western paradigms are less prominent, and the Bush Food supply is less constrained. Similarly, while some participants occasionally supplemented their diets with Bush Foods, they cited seasonality, supply instability and expense as limiting factors. There was an acknowledgement that ‘they were used a lot in the past and lots of people would eat them… but now not so much anymore because they’re hard to get’ *(P6·2, male child, inner regional).*

Participants discussed the changing food system over time and how this has affected their intake and their ability to maintain a connection to the land and engage in traditional cultural practices. Adult participants in particular expressed concerns with the effects this is having on the younger generations, describing the influence of learned behaviours from children being exposed to products such as sugary beverages and takeaway foods. The conversations alluded to two main perspectives: the role that family and community environments play and the role that built and commercial environments play in shaping dietary and health attitudes and behaviours, both underscored by an unintentional infusion of knowledge and behaviour adoption.

‘Learned behaviours. You know, you grow up, even though it was not common, but then it became more common, more affordable. So then you’re growing up in a household where, you know, you got cartons of coke sitting around. So you see that and they say you’re more likely as a child to say OK that’s normal.’ *(P8.1, female adult, major city)*

Marketing was also discussed as a key influence on participants’ food choices. Both child and adult participants spoke critically about companies purposefully utilising strategies such as sales, colours and product placement to encourage the purchasing of mostly unhealthy food, eluding that corporations were only interested in profit.

‘There’s so many unconscious behaviours we can be influenced on and we’re not even aware of it, you know. Where they position things on a shelf, and if marketed at eye height, or at children’s eye level actually is the big one, so you know, sugary things at the eye line for kids.’ *(P1.1, male adult, outer regional)*

However, several conversations also emphasised the positives that appropriate and honest marketing can bring, particularly regarding the expanding Bush Foods market. For example, a young participant noted they would be ‘more likely’ to buy a native food product if it was marketed with ‘Indigenous paintings’ *(P2·2, male child, inner regional).*

#### Bush Foods and Country create opportunities for Mob

Bush Foods were globally perceived as key to several aspects of health, with discussions highlighting the interaction of Bush Foods with Aboriginal and Torres Strait Islander Peoples’ ways of being, knowing and doing. Participants discussed, with optimism, opportunities that the growing Bush Foods and Indigenous tourism industry is bringing to communities. One child participant had parents who run an Indigenous-owned business, and her experience and enthusiasm for commercialising native food products was palpable.

‘With things like this, it usually starts as like smaller businesses, and then it makes its way up to bigger businesses. I’ve seen people try and get [Bush Food products] into airports, which is quite… usually works out sometimes… I’d like to see them brought into [main supermarket] shops a little bit more!’ *(P7.1, female child, major city)*

Adult and child participants also recognised a societal shift in opinions surrounding culture and Bush Foods, discussing ‘it’s just such an emerging market too, like the cultural stuff’ *(P1·3, male adult, outer regional).* The increasing demand for Bush Food products was discussed as an opportunity to ‘bring [non-Indigenous people] in the community a little bit… teach them about culture, because people are more open-minded’ *(P6·1, female child, inner regional)*, while also ‘improve the lives of [Aboriginal] People’ *(P5·5, male adult, outer regional)* through sustainability, nutrition, traditional medicine and economic opportunities.

However, some adult participants expressed caution and concern regarding the predominant western ownership, lack of community involvement and the commodification of Traditional Knowledge amid the growing interest in Bush Foods. Conversely, others were less concerned, provided that Bush Foods were being appreciated and utilised.

‘Like ‘Oh, that’s really good to see that this has come from here’, but there’s also a sense of ‘who’s made this’… is it a white fellas company? Or is it actual community that’s making this or have contributed.’ *(P4.2, female adult, major city)*

When discussing Bush Foods, it was evident that participants possessed significant knowledge and were open and proud to share this with the researchers. Many participants openly shared their experiences of hunting, foraging, preparing and consuming bush meats and traditional fruits and vegetables. For instance, one adult participant described their ideal lifestyle as being ‘able to walk out into [her] backyard and have forest food gardens… and go “okay what’s in season… let’s bring it inside and see what we can do with it”’ *(P8·1, female adult, major city).* Young participants were particularly enthusiastic to share their knowledge. For example, the youngest participant, P9·1, who grew up on Country in a remote community and now lives in a major city, shared extensive knowledge and stories about her culture. She outlined where to go hunting to find kangaroos and how to cook them and detailed how to identify when certain Bush Foods are ready to be eaten. She also referred to Bush Foods in language and taught the researcher how to pronounce the words correctly. Similarly, teenage girl P7·1, who also grew up on Country but in a major city, taught the interviewer how to differentiate whether a traditional plant is poisonous or not based on the leaves.

Young participants all perceived Bush Foods to provide a sense of pride and to be a valuable means of passing down ancestral knowledge and connecting to ancestors. Adult participants also discussed the cultural significance of, and emotional attachment to Bush Foods, highlighting their role beyond mere sustenance and nutrition. Participants expressed, ‘everyone is more happy when they are eating bush tucker’ (*P5·5, male adult, outer regional*), emphasising Bush Foods as a means of storytelling and connecting to Country and culture. They described the sense of belonging, pride, joy and overall emotional welfare that partaking in Bush Foods brings.

‘Yeah, well, it’s connection to Country, very much so… there’s a sense of… wow, this belongs to us, this is from our Country. This is from our land. And when I say ‘our’, it’s like all the nations together…Yeah, and also very proud.’ *(P4.2, female adult, major city)*

In addition to the emotional and symbolic significance of Bush Foods, participants emphasised their importance for physical health, expressing that ‘there’s so many medicinal purposes’ and that traditional meats such as turtle are ‘good for your blood’ *(P5·5, male adult, outer regional)*. Children also alluded to the nutritional value of traditional foods, with one young participant sharing how amazed he was at ‘how fierce warriors [his ancestors] were and how they could live off the land’ *(P2·2, male child, inner regional)*.

Participants articulated the profound impact of being on Country on individuals’ behaviours and wellbeing, with one expressing, ‘it’s being out on Country. Everyone’s happier being out on Country’ *(P5·5, male adult, outer regional)*. They viewed Country as a fundamental anchor and reflected on the intergenerational significance of Place as they discussed their families’ love for Country and its role in their wellbeing. A child participant who has recently moved from a remote community to a major city exemplified this by saying, ‘when I get home sick, I watch a bit of TV in language’ and later made a point of noting, ‘in [my Country] I don’t get sick’ *(P9·1, female child, major city)*. Returning to ancestral lands was seen as a means to reconnect with culture, find peace and escape negative influences. However, participants cautioned against viewing Country as a panacea, ‘the problem is some of these parents, they think that the Bush is a rehab clinic when it’s not’ (*P5·1, male adult, inner regional*).

#### Unwavering belief in reciprocity

Reciprocity emerged as a fundamental aspect of health and wellbeing for participants. The sharing of knowledge and resources was described as ‘communal, rather than a commodity’ *(P1·1, male adult, outer regional)*. This reciprocal exchange was viewed as a sign of respect and was perceived as a natural occurrence rather than an expectation, particularly with family, as one child participant noted ‘it’s more seen like as ‘oh, I’ll just go help them cause they’re family’, yeah’ *(P7·1. female child, major city).* Participants noted the absence of greed within their communities, emphasising that excess food was distributed throughout the community. Sharing of knowledge within and beyond the community was described as integral and seen to facilitate stronger connections, while also harbouring a sense of pride for individuals.

‘Then when we get extra crab or extra fish, we now take it to the other families. So if we don’t eat it, everyone else gets it… It’s that old trade stuff too is still how we operate, you know that sharing with this mob and that mob… that sort of old system – it’s sort of disappearing, but there’s a lot of value in that sort of stuff – that connection to other people, to other family.’ *(P1.3, male adult, outer regional)*

Young participants also echoed the importance of reciprocity, describing, ‘giving back to the community… I reckon it would be pretty good cause then it means there’s more food for people to share around’ *(P6·2, male child, inner regional)*. In fact, reciprocity was extended beyond family and kin to the natural world and Country. Participants described that this reciprocal nature was mutually beneficial.

‘So being able to care for plants… Yeah, I actually benefit from their energy. They benefit from my care and then they produce and I get to enjoy that. And so the ultimate outcome is being able to partake from [Country].’ *(P8.1, female adult, major city)*

Participants also described the continual transfer of knowledge within families and through generations. Child participants described their parents and extended families embedding Traditional Knowledge unto them. P9·1, who held vast Traditional Knowledge and had the ability to speak four Aboriginal languages at only 11 years old, described that her knowledge had been passed down through at least three generations. Child participants also discussed caregivers taking them to Country to teach them about plants and Bush Foods.

‘We will go for a walk for a while and Mum might stop on the side of the path and she might walk in a little bit and she’ll find like a fruit or something – tamarind! And she’ll go “Oh, here look, you can eat this” and then she can tell you about what you can do with it if it has any healing properties like anything like that and it’s quite interesting to see.’ *(P7.1, female child, major city)*

This intergenerational knowledge transfer was regarded as vital by participants and perceived as a crucial facilitator of positive health behaviours and a protective psychological factor. Participants noted that families not engaged in such knowledge exchange often experienced poorer health outcomes.

‘But because Nana and Granny got everyone off [the mission] and then straight back into hunting and fishing and stuff our family is not too bad. But when we look at other families around us, it’s worse.’ *(P1.3, male adult, outer regional)*

#### Agency v non-agency and links to health behaviour

The concept of individual choice and agency emerged as a significant determinant of nutrition and health. While acknowledging the influence of external factors on choice, participants primarily attributed the responsibility for change to the individual. Agency was closely linked to health literacy and education, with informed decision-making perceived as integral to maintaining health. Participants demonstrated a high level of health literacy, critically evaluating nutrition-related information and making informed choices. For instance, they expressed scepticism about the Australian health star rating system and deceptive advertising practices regarding ‘low-fat lollies’ *(P1·1, male adult, outer regional)*, showcasing a nuanced understanding of nutrition.

This sophisticated level of health literacy extended to child participants, who recognised the importance of healthy living for creating ‘more opportunities than being unhealthy’ (*P2·2, male child, inner regional)* and preventing chronic diseases. They exhibited awareness of familial health patterns, such as diabetes ‘runs in [the] family’ *(P3·2, male child, inner regional)*, and displayed knowledge of bodily functions and medical conditions, demonstrating a keen understanding of health concepts beyond their years.

Participants recognised that rational decision-making could be influenced by emotions and personal preferences, particularly when it came to food choices. Despite being aware of the health implications, participants discussed occasionally opting for unhealthy foods like takeaway due to taste preferences, convenience or emotional reasons. This interplay between rationality and emotionality was succinctly captured by one participant’s statement: ‘I know what I should do, but I’m an emotional eater’ *(P4·2, female adult, major city)*.

Participants perceived education as a partially self-driven process, intertwined with self-care and self-worth, which could have profound ripple effects on families and communities. This ripple effect often extended across familial lines, with the agency of parents directly impacting their children. Participants highlighted the case of a mother whose health behaviours were hindering her son’s achievements, noting that ‘her own issues are the barriers’ *(P5·4, female adult, outer regional)*. This contrasted sharply with a participant who had actively chosen to prioritise their family’s education and health.

‘But if I care about me then, well, I make an effort to do things differently, and so will I for my children. So if there’s that absence of education and the absence of self-care, then they’re even more at risk.’ *(P8.1, female adult, major city)*

Participants expressed a continuous yearning for knowledge about nutrition, Bush Foods and culture, with this desire particularly evident among child participants. Child participants articulated a desire to learn more about Bush Foods as a means to ‘connect more with Aboriginal People and Torres Strait Islanders’ *(P2·1, male child, inner regional)* and ‘to see what Elders tasted and to learn about the things they used to eat’ *(P2·2, male child, inner regional).* This yearning for learning was also observed by adult participants, who noted ‘there’s heaps of kids out there that want to learn [about culture]’ *(P5·1, male adult, inner regional)*.

## Discussion

**Figure 2.**
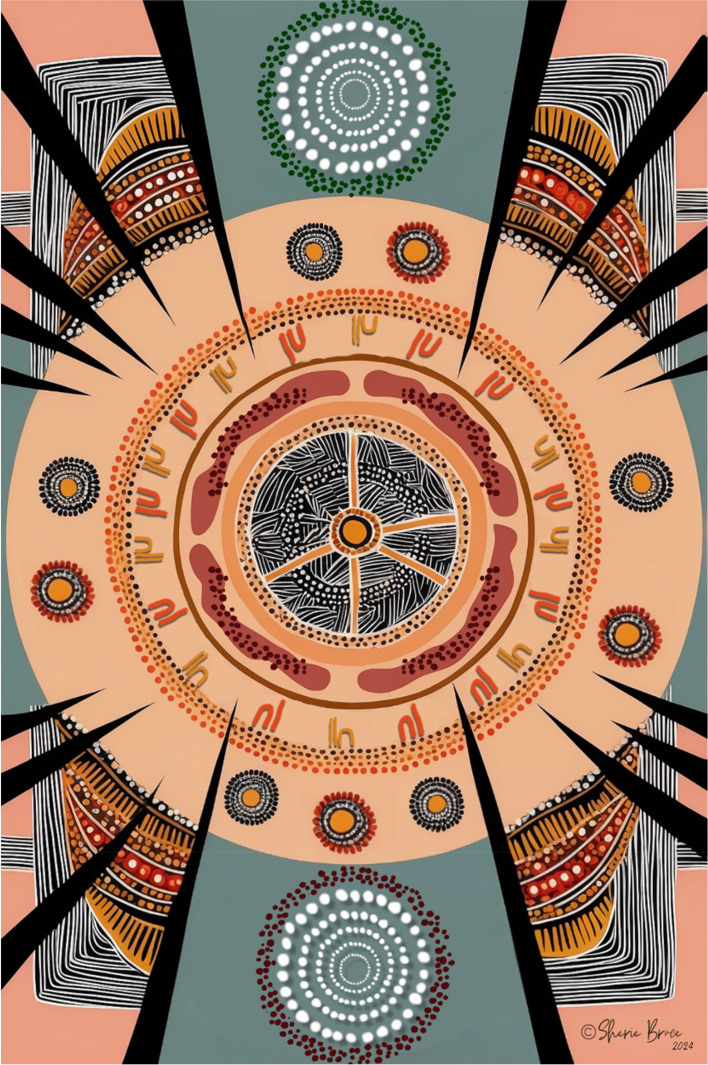
An artwork titled ‘Bush Foods *v* Capitalism’ created by author S.B., a Yolngu and Arrente woman, visually representing her interpretation of the results. Disclaimer: This image was created with assistance from artificial intelligence, with careful attention to upholding cultural integrity and respect (see Supplementary Material 3 for further details). Caution must always be exercised when using artificial intelligence to generate art, particularly to ensure the protection of intellectual and cultural property.

The present study aimed to explore the experiences, perspectives and insights of urban and regional living Aboriginal and Torres Strait Islander adults and children regarding Bush Foods, nutrition and health. The perspectives of participants were multi-dimensional and interconnected, aligning with the holistic concept of health as including physical, social, emotional, spiritual and ecological wellbeing held by Aboriginal and Torres Strait Islander Peoples^(5,42)^. This finding is consistent with similar research on the topic and strengthens the knowledge base that privileges Aboriginal and Torres Strait Islander voices^(2,37)^. Through the thematic analysis process, five key themes were generated: the force of colonisation eroding health; the socio-environmental conditions with a strong hold on food choice; opportunities created through Bush Foods and Country; a global, unwavering belief in reciprocity; and the role and nuances of agency and choice regarding health. The emergent themes were highly interwoven and overlapping, yet they can be synthesised and summarised into two key overarching concepts discussed in existing literature: at the individual and community-environment level – the role of family, kin and culture, akin to the previously established cultural determinants of health^(5)^; and at the broader socio-political level – the influences of colonialism, capitalism and imbalances of power, akin to the previously identified social determinants of Indigenous health^(43,44)^. As shown in Fig. 2, the First Nations author of this paper has crafted an image to represent her interpretation of the findings within this study. Refer to SM3 for a detailed explanation of the meaning behind this artwork and how it was created.

The findings of this research indicate individual and community factors such as family, kin, community, culture and Place (Country) were identified as protective factors for health and nutrition, consistent with previous research on these cultural determinants of health^(5)^. This underscores the need for policy reforms that respect Indigenous rights and support community self-determination. Importantly, child participants in the study also recognised these cultural determinants, validating the importance of cultural determinants of health for Aboriginal and Torres Strait Islander People across the lifespan. Conversely, socio-political factors, such as colonialism, capitalism and imbalances of power, were perceived as detrimental to participants’ health and nutrition. This aligns with previous research discovering these broader systemic and structural factors, such as government structures, policies and socio-economic positioning, referred to as the social determinants of Indigenous health, are critical in influencing health outcomes^(43,44)^. In the present paper, these factors were linked to disrupted access to Country and culture, racism, food insecurity and intergenerational trauma. The impact of these negative influences was described as having a ripple effect on the community, ultimately undermining the aforementioned cultural determinants of health. Interestingly, these factors were discussed quite emotively by adult participants who have decades of lived experience to draw on, whereas it appeared child participants were sheltered from or not yet cognisant to some of these harsh realities. This suggests that the environment, rather than individual choice, predominantly governs health decisions, highlighting how western ideals have imposed on Aboriginal and Torres Strait Islander Peoples’ engagement with culture. The profound impact of historical traumas on contemporary Aboriginal and Torres Strait Islander Peoples’ experiences highlights the urgency for initiatives aimed at facilitating cultural revitalisation and healing.

The results of our study validate the cultural determinants of health^(5)^ and social determinants of Indigenous health^(43,44)^ through the lens of lived experience. These determinants have been recognised and incorporated into the development of the NATSIHP 2021–2031^(9)^. Building on its previous version, the NATSIHP 2021–2031 is an Australian national policy directive aimed at guiding the development of all Aboriginal and Torres Strait Islander health policies, programmes and initiatives over the next decade^(9)^. Developed in consultation with the community and grounded in the foundations of cultural and social determinants, the NATSIHP represents a positive departure from the historical trickle-down policy. However, despite being released for over 2 years, and its framework promising accountability, there is currently no evidence of implementation or reports of outcomes. This policy silence suggests that the same issues of suboptimal implementation and evaluation, evident in the Close the Gap policy and similar initiatives, persist^(10)^.

Previous policies, despite their well-meaning intentions, have consistently failed to ‘close the gap’ in health outcomes between Aboriginal and Torres Strait Islander Peoples and non-Indigenous Australians^(45)^. This may be due to majority of previous health initiatives targeting Aboriginal and Torres Strait Islander communities emphasise individual choice and responsibility as the primary drivers of health outcomes^(12,46,47)^. Consequently, policy failures have often been perceived as the failure of the people, rather than the failure of the policies themselves^(47)^. In reality, these failures are associated with a lack of ‘insider knowledge’ – an understanding of community priorities and appropriate engagement with Aboriginal and Torres Strait Islander ontologies and epistemologies^(47)^. As our results illustrate, policies that impose western worldviews on Aboriginal and Torres Strait Islander Peoples do little to address disengagement and distrust in government. This underscores the necessity for Aboriginal and Torres Strait Islander ways of knowing, being and doing to be central to planning, delivery, implementation and evaluation of health policies, programmes and services^(46,47)^.

In the context of our study’s focus on Bush Foods, nutrition and health, it is striking to note the lack of both national and state-level policies addressing the specific food and nutrition needs and desires of Aboriginal and Torres Strait Islander populations in Australia. This oversight is particularly noteworthy given the absence of any mention of nutrition or Bush Foods in both the NATSIHP and the Close the Gap strategy^(8,9)^. Previous scholarly works suggest this omission of targeted food and nutrition policy may reflect the complexities of nutrition; however, it is more likely indicative of a systemic lack of political will, combined with a lack of opportunity for Aboriginal and Torres Strait Islander leadership in policy-making^(45)^. The findings of our study, emphasising the crucial role of Bush Foods and nutrition in health, underscore the pressing need to rectify this policy gap by prioritising the amplification of Aboriginal and Torres Strait Islander voices and promoting inclusivity at the policy table. Moreover, this study contributes to the growing body of research suggesting the imperative to ‘modify structures and systems governing food supply through improved availability, access and affordability of healthy foods’^(45)^. Specific to urban-living Indigenous Peoples, the findings of this paper regarding tensions of existing in a colonised environment and yearning to be more closely connected to culture highlight the necessity of developing a culturally informed national nutrition strategy tailored to this population. Combined with increased deregulation of access to traditional foods for Aboriginal and Torres Strait Islander Peoples, such efforts are crucial.

The 2023 Food Policies for Aboriginal and Torres Strait Islander Health (FoodPATH) project^(48)^ is a noteworthy example of culturally informed transformative policy initiatives. It employed a ‘systems dynamic approach’ to privilege the voices of fifty-three Aboriginal and Torres Strait Islander participants. These participants identified individual, community and systemic factors influencing food choices such as family, community and culture, nutrition education, limited access to healthy foods and increased access to unhealthy foods, aligning closely with the findings of our present study^(48)^. The researchers collaboratively formulated recommendations that emphasised not only community-driven actions but also the necessity for broader systemic government strategies to reshape the food environment, which the results of our study reiterate to be integral. This approach to policy reform shifts the responsibility solely from individuals and communities to a truly shared responsibility with those in positions of political power. Such a shift in focus from individual-based to community and systemic-based strategies offers a vision for how future policy can positively influence health behaviours and nutrition among Aboriginal and Torres Strait Islander Peoples and communities. These recommendations align with an incremental, culturally led approach to policy transformation, which will also serve to remove the unnecessary ‘red tape’ described by participants in our study.

A key finding from this research that has been echoed by previous research^(4,48)^ is that incorporating traditional Bush Foods into modern diets strengthens cultural identity and promotes health. Therefore, to assist urban-living Indigenous People in connecting to their culture where evidence of colonisation is present with every turn, we recommend governments develop a culturally informed health policy which supports and funds Indigenous-led nutrition programmes that integrate Bush Foods into schools, community centres and healthcare settings. This may include providing resources for community-led cooking programmes, incorporating Bush Foods into public health nutrition guidelines and ensuring Indigenous enterprises have viable pathways to commercialise and distribute these foods. These small but pragmatic steps create culturally meaningful health initiatives while laying the groundwork for broader policy shifts in the future.

A unique finding from this study is that when working within an urban-specific context, there was much more interest around this commercial perspective. Participants enthusiastically discussed opportunities for Mob to use Bush Foods to generate enterprise and income. This is complemented by previous literature exploring the commercial determinants of Indigenous health and how, if harnessed appropriately, they can have positive impacts^(49)^. However, a different school of thought may consider this ‘commodifying of culture’ as fundamentally incongruent with traditional Aboriginal lifestyles^(50)^. The reality is that most urban-living Indigenous People exist within a post-colonial capitalist society not designed for them, meaning creativity, adaptation and resilience are required to thrive. This research has highlighted the frustration and distrust felt by participants towards the food industry and the government, exemplifying how commercial activities can be to the detriment of Indigenous health^(49)^. The Australian government must take an active role in challenging powerful commercial forces by holding industry accountable for the role they play in Australian health inequity. This could be done through mandating food and beverage companies to undergo Indigenous-led health impact assessments.

### Strengths and limitations

A significant strength of this study lies in the inclusion of a diverse range of participants, spanning different age groups, genders and geographical locations. This diversity enabled an exploration of health and nutrition perspectives across the lifespan, from childhood to adulthood. However, it is important to note that these findings are context-specific and may not be universally applicable to all Aboriginal and Torres Strait Islander communities. Therefore, while the study provides valuable insights, ongoing place-based engagement with communities is essential to ensure a comprehensive understanding of each community’s diverse health and nutrition needs and priorities.

The research aimed to adhere to the guidelines set forth in the CREATE tool^(27)^ and the CONSIDER statement^(26)^. While the study demonstrated a commitment to cultural sensitivity, involved Aboriginal and Torres Strait Islander researchers and was designed in response to priorities outlined by UAF’s Indigenous partners, one of the primary constraints was the inability to fully implement a participatory action research (PAR) methodology, which is highly valued for its inclusive and collaborative nature^(28)^. The inability to adopt a PAR approach was influenced by several factors, including time constraints, limited financial resources and logistical challenges. While the study was culturally responsive and did consider elements of PAR, it fell short of the ideal of being an entirely Indigenous research team or by having participant involvement in the research design, process and outcomes. The involvement of the majority of non-Indigenous researchers, while supportive, does not fully align with the gold standard for conducting research that is deeply rooted in the community’s own governance and knowledge systems^(27)^. Future studies should aim to overcome these barriers by securing adequate resources and adopting frameworks such as PAR to ensure that research is not only culturally relevant but also directly beneficial to participants, promoting a more impactful research process.

## Conclusion

Our study elucidates the nuanced perspectives of Aboriginal and Torres Strait Islander People living in major cities and regional areas on Bush Foods, nutrition and health, highlighting the interconnectedness of cultural, socio-political and individual factors. Importantly, these findings were disseminated to the UAF First Nations Enterprise Group as it offers valuable insights in informing them as they work to build an Indigenous-led native foods sector. More Indigenous-owned Bush Food businesses will assist with financial stability within communities and increase the availability and accessibility of Bush Foods, alleviating current concerns raised by urban-living Indigenous participants. Our findings underscore the urgent need for culturally informed nutrition policies that privilege Indigenous voices and promote self-determination. Policies should not only be developed in consultation with Aboriginal and Torres Strait Islander communities but also led by them, ensuring that they reflect Indigenous values, knowledge and priorities. By addressing gaps in policy implementation and amplifying Indigenous perspectives, the findings from this research advocate for an Indigenised food system where Traditional Indigenous Knowledge, foods and practices are integrated into contemporary food production. This not only enhances the wellbeing of Aboriginal and Torres Strait Islander Peoples but also facilitates inclusivity and sustainability for all Australians. It offers a counter-narrative and a way to shift the food system towards Indigenous ownership and benefit. Prioritising cultural determinants of health and integrating them with social and commercial determinants is essential for developing effective health strategies. Future research and policy efforts must continue to engage deeply with Indigenous communities, ensuring that their insights and leadership guide the path towards better health outcomes.

## Supporting information

Cartwright et al. supplementary material 1 Cartwright et al. supplementary material

Cartwright et al. supplementary material 2 Cartwright et al. supplementary material

Cartwright et al. supplementary material 3 Cartwright et al. supplementary material

## Supplementary material

For supplementary material accompanying this paper, visit https://doi.org/10.1017/S1368980025100694.
