# Supplementary material for: Exploring the perspectives of urban and regional living Aboriginal and Torres Strait Islander Peoples regarding bush foods, nutrition and health: insights for culturally informed health policy in Australia
Source: Public Health Nutr. 2025 Jul 17;28(1):e124. doi: 10.1017/S1368980025100694 (PMC12465064; doi:10.1017/S1368980025100694)
Supplement: Cartwright et al. supplementary material 1 — Cartwright et al. supplementary material [file S1368980025100694sup001.docx]

Positionality of the Research Team

## Relevance

The specific approach our research team has taken seems to have not been done before in the qualitative space, hence wanting to include this supplementary material to offer further detail with regards to our methodological choices. We wanted a space for us to detail some of this decision-making as it cannot be included within the main text of the manuscript due to word limit constraints – which is why we have created this additional document. Ensuring theoretical and methodological coherence in our paper has been front-of-mind throughout the entire process, and we do believe there is appropriate rationale for each decision we have made, given the research teams’ resources, experience, and positionality.

## Research Aim

Explore the experiences, perspectives, opinions, and insights of urban and regional living Aboriginal and Torres Strait Islander adults and children regarding Bush Foods, nutrition, and health.

## Research Team

First Nations academics (T.B., K.W., S.B.) and J.C. were involved in conceptualising the Yarns and planning the data analysis process. S.B., K.W., T.B., J.C., Y.S. and the Uniquely Australian Foods (UAF) First Nations Enterprise Group Chair were involved in participant recruitment. J.C. and S.B. were involved in data collection. Two non-Indigenous researchers (N.T., J.C.) and one Indigenous researcher (S.B.) were involved in data analysis, with non-Indigenous researcher O.W. assisting S.B., N.T. and J.C. in codebook creation. The UAF First Nations Enterprise Group Chair assisted in ensuring culturally respectful conduct and addressing of research priorities. Two non-Indigenous researchers (Y.S. and M.N.) were involved in a supervisory capacity.

Please see below a detailed positionality written by each of the authors: J.C., N.T., S.B., Y.S., M.N., and O.W.

### Jessica Cartwright

I am an Accredited Practising Dietitian (APD) and a PhD scholar at the University of Queensland (UQ). I have European heritage but have not been deeply connected to my cultural roots. In fact, this research has inspired me to explore my own ancestry, and I plan to visit my family in the Czech Republic after completing my PhD to learn more about my culture. This personal journey reinforces my appreciation for the significance of cultural identity and the role that Traditional Knowledge and connection plays in health and wellbeing.

My research is part of the UAF Training Centre, directed by Professor Yasmina Sultanbawa, who is highly regarded by the centres’ Indigenous partners for her kind and empathetic approach and her extensive experience in the native foods space over the past decade. Yasmina plays a key role in maintaining ongoing engagement with the centres’ Indigenous Advisory Committees. Looking at Yasmina’s relationship with the Indigenous communities involved in the centre, I would describe it as more akin to family rather than work partners. My PhD focuses on working with Indigenous communities to co-create a native food-flavoured, low sugar, healthier alternative to a regular soft drink. This project aims to support connection to Country and improve health outcomes for Indigenous People by reintroducing Bush Foods into contemporary diets in a way that aligns with cultural values and preferences.

While I am technically the ‘lead researcher’ in this project, I do not see this as reflective of the research process itself. Therefore, I am hesitant to refer to myself as this as I feel like it lessens the contributions from the broader research team. Rather, I believe all members of the research team led the research in different ways. Throughout the research process we all spoke fondly of the idea of us ‘sharing a brain’ and there was always utmost respect for each researchers’ area of expertise.

Through this research, I have had the privilege of facilitating Yarning circles alongside S.B. with Indigenous adults and children, discussing perspectives on Bush Foods, nutrition, and health. Despite approval from the First Nations Enterprise Group Chair and S.B., I spent a lot of time considering the appropriateness of myself, a non-Indigenous researcher, as a facilitator of the Yarning circles. I reflected on this in the context of insider/outsider discourse. I was not a complete ‘outsider’ in these Yarns – I had prior connections with majority of the participants, I live in an urban area which is similar to all participants, I have similar beliefs, worldviews and socio-political views to many of the participants, and my day-to-day responsibilities are/have been similar to many of the participants. Of course, that does not make me an ‘insider’ in every regard – and there are obvious aspects where I am considered an ‘outsider’ (e.g. I do not have lived experience of what it feels like and means to be an Indigenous Person living in Australia). I acknowledge that in a different cultural context – such as a remote Indigenous community – I would lack the necessary insider knowledge to facilitate Yarning, and it would not be appropriate for me to do so.

I have been involved in Indigenous health research for four years. Within this time, I have made strong relationships with remote and urban-living Indigenous People and communities. I have worked with remote schools to deliver cooking classes incorporating Bush Foods, as well as co-design and deliver healthy eating workshops for students with community Elders. I have also volunteered at several urban Indigenous school events. I have undertaken extensive cultural training through UQ and participated in Indigenous methodology Yarning sessions hosted within the Queensland Alliance for Agriculture and Food Innovation (QAAFI) to deepen my understanding of Indigenous ways of knowing, being, and doing.

Beyond my cultural training and experience, I bring high-level technical skills and knowledge in nutrition and qualitative methodologies. Before embarking on this research project, I read entire textbooks on qualitative research, did a thorough review of the literature regarding methodologies and common oversights or missed opportunities in qualitative research, and had email correspondence and meetings with highly esteemed qualitative experts and researchers to talk through theoretical and methodological tensions that may arise in this project. I would describe myself as an ambitious and determined researcher, but I also recognise that self-awareness and humility are among my greatest strengths. Entering this research, I understood how much I needed to learn about conducting qualitative research within an Indigenous research paradigm. I approached this work with deep respect, a willingness to listen, and an openness to being guided by Indigenous voices.

### Niall Turner

I am a health care worker with a passion for Bush Foods and Indigenous health. With a background in ecology, natural areas management, and dietetics, I am passionate about Indigenising the Australian food system to combat health injustices and climate change. My work is grounded in both scientific research and community collaboration, ensuring that Indigenous voices and knowledge are central to shaping sustainable food systems. Ultimately, I want to create a better future for *all* Australians.

I am of Sicilian heritage, coming from a long line of peasant farmers who emigrated to Australia for better opportunities. Growing up with a deep appreciation for the land and traditional foodways, I understand the importance of preserving cultural food knowledge and ensuring food sovereignty. My heritage has contributed to my passion for land management, food system reform, and social and nutritional equity in the context of a westernised food system which largely ignores the value of Indigenous cultivation and food provision practices.

### Sherie Bruce

I am a mature-aged, 52-year-old female Aboriginal scientist, consultant, researcher, and strategist with a background in environmental science, mycology, microbiology, and biotechnology. I was born and raised in remote communities of Arnhem Land and have deep cultural connections to the Arrernte and Yolŋu Nations of the Northern Territory (NT). I have lived and worked in several remote and regional towns across the NT. I have witnessed firsthand the challenges and strengths of Aboriginal communities navigating government frameworks and scientific institutions.

Since my twenties, I have struggled to achieve my dream of becoming a scientist, a goal I finally realised in my forties. Despite working in sectors that acknowledge the importance of Aboriginal perspectives, I continue to experience the broader systemic challenges that many Indigenous researchers face in academia. The struggle for authentic Aboriginal leadership, inclusion, and decision-making power in research is ongoing, and I remain committed to advocating for meaningful change. My work spans Aboriginal inclusion, strategy, environmental science, and business sectors, where I continue to push for Indigenous-led decision-making and systemic transformation. As a team member of the Commonwealth Scientific and Industrial Research Organisation’s Tiwi People’s prawn farm project, I contribute rigorous Indigenous research methodologies to ensure culturally responsive and impactful outcomes. Through all of my work, I aim to create meaningful change where Aboriginal perspectives are embedded in all sectors of Australia, not as an afterthought but as the foundation. I continue to fight for a future where Indigenous methodologies are standard practice, Aboriginal People lead research on issues that impact them, and where partnerships built on trust, respect, and reciprocity drive meaningful outcomes for our communities.

I am a PhD scholar currently studying Aboriginal mycology in the Training Centre for UAF under QAAFI, supervised by Professor Yasmina Sultanbawa. Professor Sultanbawa has extensive experience working with Aboriginal People in the Bush Food space, particularly in ensuring Indigenous voices and priorities are respected in research, industry development, and food science. Her work has contributed significantly to recognising Aboriginal Bush Foods in mainstream food systems while advocating for ethical engagement and knowledge protection. I understand this research was reviewed by UAF’s First Nations Enterprise Group and the First Nations Advisory Group, ensuring that the approach aligned with Indigenous priorities and ethical considerations. Their oversight was instrumental in reinforcing the study’s commitment to Aboriginal leadership, cultural integrity, and knowledge sovereignty.

I strongly believe in reciprocity, and in this research led by Jessica, we worked the ‘right way’ (Aboriginal Traditional way of side-by-side with deep respect and genuine sharing), and every effort was made to privilege Aboriginal voices and respect their decisions. Jessica, as a non-Indigenous researcher, was cautious about conducting Yarning so as to not overstep a cultural boundary. Still, I affirmed that it could be done appropriately in partnership with and guidance from Aboriginal researchers (T.B., K.W., and myself). I firmly believe that when done the ‘right-way’, non-Indigenous researchers can contribute to increasing the visibility, inclusion, and privileging of Indigenous methodologies, ultimately ensuring they lead the research process. I worked closely with Jessica to prepare her for the Yarning sessions with participants, ensuring she understood the depth and cultural responsibilities associated with the methodology. I attended most of the Yarning sessions, observing how she engaged with participants. Throughout these sessions, I felt confident she held to the Yarning methodology, demonstrating respect, active listening, and a genuine commitment to amplifying Aboriginal voices. I was 100 % comfortable with her approach and trusted her in this role. This trust was further reinforced when I listened to each recording of the Yarning sessions during the coding phase, where it became evident that the integrity of the method had been upheld.

In this study, my positionality has been critical in shaping the approach to Aboriginal priority, inclusion, and engagement. I bring a decolonial and systems-thinking lens to my work, ensuring that Indigenous perspectives are embedded in research in ways that respect cultural authority, knowledge sovereignty, and self-determination. My skillset lies in providing project design advice that ensures the respect of, and embedment of the values, and cultural protocols of Aboriginal and Torres Strait Islander Peoples, as well as providing advice on culturally respectful ways of working with Aboriginal adults and children. Therefore, I have ensured to the best of my ability with my knowledge, experience and skills that the entire research process was undertaken in a culturally appropriate manner. I am extremely grateful to have been involved in this research – it has been an incredible experience as it has allowed me to extend my technical skillset as an Indigenous researcher, which I look forward to applying to future projects.

### Yasmina Sultanbawa

I am a food scientist with over 20 years of research experience. My passion is to bring food science to the forefront in addressing food and nutrition security, two of the biggest challenges facing an increasing global population. I have been successful in using food science as a tool to provide health and equity for marginalised communities. My work in this area started in Sri Lanka, addressing the issue of protein malnutrition and working with remote fishing communities to create social enterprises where science and technology were used to develop value-added fish products. This work was centred around empowering women in playing a lead role in establishing these enterprises. When I migrated to Australia 12 years ago, I drew a parallel to my work in Sri Lanka when I saw a similar situation in Indigenous communities in Australia, where lack of a balanced diet containing foods rich in vitamins and minerals was having adverse health and socio-economic impacts. I saw an opportunity to research the chemistry of endemic Australian flora and use that knowledge to develop a class of ‘Uniquely Australian Foods’. I now work with Indigenous communities’ across Australia, and I was the first researcher to introduce and implement the idea of using native plant foods as natural ingredients that provide health value or other specific properties in food industry applications.

Coming from Sri Lanka, I understand and relate to lots of the marginalisations experienced by First Nations People living in Australia. This unique bond I feel I intrinsically have with Aboriginal and Torres Strait Islander People makes me believe I was always destined to work in this field. UAF was then established in 2019 with Australian Indigenous partners with a shared vision of creating an Indigenous-led native foods sector and reintegrating native foods (through value-adding) into the food system. Despite commencing in 2019, my relationships with the Indigenous partners pre-date this by several years. Within my role as director at UAF, I have coordinated a suite of research with PhD students, Post-docs, and Chief Investigators across a range of different areas; food science, nutrition science, law, social science, health translation, and so on, in an attempt to make this vision become a reality. My current research endeavours include investigating the application of blockchain technology to ensure traceability of Indigenous-owned food products and exploring how Traditional Knowledge held by communities can be harnessed to improve their health. The research outputs and findings from UAF are relayed back to the Indigenous partners to inform their approach to nutrition, health, and business.

### Michael Netzel

My research background is in the field of nutrition and food science. I am a Senior Research Fellow within CNAFS under QAAFI and a Chief Investigator within UAF. I have been working with native foods since 2005 and started working at UAF in 2019. I have several years’ experience in engaging with First Nations partners and communities, making myself well-versed and skilled to contribute to this field. Recently, I have been exploring the nutritional properties of halophytes and how this Bush Food could be used as a salt substitute.

I am from Germany and currently live in Australia with my family. I understand the importance of maintaining a connection to one’s culture as I ensure my two daughters remain connected to their German roots through food and language.

### Olivia Wright

I am Dr Olivia Wright – of Irish/Scottish descent, born and raised in Australia. I am deeply committed to advancing Indigenous-led research in health and Bush Foods. As an Advanced APD, an Affiliate Senior Research Fellow at the Centre for Nutrition and Food Sciences within QAAFI, and a Senior Lecturer in Nutrition and Dietetics at UQ, I have devoted my career to understanding the intricate relationship between diet, health, and disease. My longstanding involvement in First Nations health research is built on a foundation of compassion and cultural sensitivity, ensuring that every interaction with Indigenous communities is both respectful and safe.

For over 15 years, I have taught cultural competence and cultural humility to Master of Dietetics students, equipping emerging professionals with the skills necessary for thoughtful and collaborative practice. My journey in cultural understanding has been enriched by completing BlackCard cultural training and Indigenous Allied Health Australia cultural training. These experiences have not only deepened my respect for Indigenous perspectives but have also reinforced my commitment to embedding these values in both my research and teaching.

Since 2019, my role with the Training Centre for UAF has opened doors to unique collaborative partnerships with Indigenous industry leaders in the Bush Foods sector. I have had the privilege of working alongside elders from across Australia, engaging with communities in the NT on nutrition and food, and co-delivering educational sessions to young Indigenous People through initiatives with Cricket Australia and the Murri School in Brisbane. These experiences have underscored for me the transformative power of community-driven research and the importance of integrating Traditional Knowledge with contemporary scientific inquiry.

In addition to my research and community engagement, I have actively contributed to curriculum reform at UQ by participating in the Indigenising Curriculum Working Party and driving change as Director of Teaching and Learning within my school. Over the past two years, I have worked to ensure our curricula honour the histories, cultures, and contributions of Indigenous Peoples, advocating for culturally responsive teaching methods that prepare our students for respectful and informed practice.

My passion for Indigenous-led research fuels everything I do. I am committed to continuously learning from and collaborating with Indigenous People to advance our collective understanding of health, nutrition, and the rich heritage of Bush Foods. By centring Indigenous voices and knowledge, I believe we can foster a research and educational environment that is both inclusive and transformative.

## Terminology

Throughout our manuscript we have purposefully chosen to use different terms such as ‘Bush Foods’, ‘Traditional Foods’, and ‘Native Foods’ as we believe it is crucial to reflect the diversity and richness of Aboriginal cultures and languages. Each term carries its own nuance and significance, which is important to preserve in academic writing.

Similarly, we have intentionally used both ‘Indigenous’ and ‘Aboriginal and Torres Strait Islander’, as well as ‘First Nations’ throughout the manuscript to reflect the diversity and distinct identities of Australia's First Nations Peoples. This choice is deliberate, as it acknowledges the unique cultural and historical contexts of each group. We do not want to homogenise our writing.

Finally, our choice to use a lowercase ‘w’ for ‘western’ is deliberate, reflecting our commitment to decolonising academic language and challenging traditional hierarchies. This approach acknowledges and respects diverse knowledge systems, including Indigenous perspectives, by not automatically prioritising western paradigms.

## Research Approach

Every decision made surrounding theoretical lens and methodology was considered in the context of two main objectives:

1. Centring Indigenous voices and privileging Indigenous knowledge
2. Obtaining high quality research results that will benefit Indigenous People

We did not enter this research project with a predetermined approach or method in mind, but rather from a perspective of curiosity and genuine interest in ensuring the best possible approach to achieve those two objectives. Of course, this had to be considered in the context of funding, resources, logistics, research team member capacity and skillset, timeframes and deadlines, and so on. We searched published literature to find a methodology used in a previous study that would ‘fit’ our pragmatic considerations while aligning with our two objectives but were unable to do so. Therefore, we explored all the possible approaches we could take (e.g. attended online seminars, read textbooks, literature searching, talking to people, etc.), and considered them in the context of how it aligns with our two objectives. Rather than trying to adjust, bend, or fit our approach to something done previously, we made a conscious decision to steer in the direction of innovation and use a hybrid approach. Grounded in elements of pragmatism, this allowed us the flexibility to maximise the fit and alignment of our research purpose, context, and constraints.

To allow our research aim to be achieved we required a more experiential orientation, and a more semantic interpretation of the data to allow the voices of our participants to be truly privileged and limit the extent to which the non-Indigenous researchers are interpreting the data throughout the analysis process. Therefore, we adopted a critical realism ontology, which aligns with many Indigenous ways of knowing, such as:

- Recognition of multiple truths – Critical realism acknowledges that while an objective reality may exist, our understanding of it is shaped by experience, interpretation, and social context. This aligns with Indigenous epistemologies that value relational knowledge and plurality of perspectives.
- Respect for lived experience – The Yarning process privileges the lived experiences and stories of Indigenous participants, which aligns with critical realism’s commitment to acknowledging subjective realities while understanding underlying social constructs.
- Social and cultural context – Indigenous methodologies emphasise connectedness, relationships, and the impact of historical and cultural structures on knowledge. Critical realism similarly considers how deeper social realities influence individual experiences.

We selected Yarning as the most appropriate data collection methodology because it originates from Indigenous cultural practices and fosters a culturally safe, participant-led process. Grounded in relationality, storytelling, and flexibility, Yarning allows participants to drive the conversation in directions that are most meaningful to them. This approach aligns with our experiential research aim – to explore the perspectives and experiences of Indigenous People regarding Bush Foods, health, and nutrition. By using Yarning, we were able to generate richer, more authentic insights compared to structured methods such as interviews or focus groups, which may have resulted in short, rigid responses to pre-determined questions (this is again in accordance with our aforementioned research objectives). By combining Yarning methodology with a critical realist ontology in a pragmatic way, we were able to capture data that reflects both the real experiences of participants and the contextual factors that shape those experiences.

We believe our research sits somewhere in between the ‘small q’ and ‘big Q’ qualitative frameworks discussed by Braun and Clarke (2022). First Nations’ author (S.B.) made an interesting comment that we spent some time mulling over and reflecting upon – this approach ‘goes against’ the ‘Indigenous way’ of doing research. This put us in a strange paradox where we felt as though our approach needed to be more ‘western’ to allow for higher quality findings to be obtained from our Aboriginal and Torres Strait Islander participants. We recognise that Indigenous worldviews are often grounded in relativist ontologies. However, we believe critical realism provided a pragmatic and rigorous analytical framework that allowed us to both privilege participant narratives and acknowledge the interpretative role of the researchers. A relativist ontology, while conceptually aligned with Indigenous epistemologies, was deemed methodologically impractical due to constraints in coder consistency and project feasibility (i.e. time and budget constraints). Best practice suggests a single coder for relativist analysis which was not viable nor appropriate as it may map broadly to a more critically-oriented research question and latent interpretation of the data – which could create methodological incongruencies. Critical realism provided a structured yet flexible approach that enabled us to achieve our two objectives: balance methodological rigour while upholding ethical responsibility of privileging Indigenous knowledge.

We were able to respect participant narratives as ‘truths’ while acknowledging that knowledge is socially and culturally situated. To strengthen Indigenous voices, we ensured that SB – a researcher with expertise in Yarning methodologies – played a central role in the coding process. This helped to prioritise Indigenous knowledge and perspectives while maintaining methodological consistency across multiple coders (particularly given that two of three coders were non-Indigenous). We recognise that an experientially-oriented research question aligns broadly with a semantic interpretation of the data. Our decision to adopt a semantic approach was reinforced by our commitment to centring Indigenous voices and producing high-quality, meaningful research outcomes. A critical realist ontology meant we were able to ensure consistency across coders and maintain methodological rigor to facilitate a more semantic interpretation of the data.

## Research Method

We conducted nine audio-recorded Yarns with a total of 20 Indigenous participants (10 adults, 10 children). T.B. provided four key insights on ethical Yarning practice, all of which were upheld throughout the study: 1) Give back, do not just take, 2) Build relationships – interact for non-research related purposes 3) Ensure sessions are conversational, 4) Privilege Indigenous knowledge – centre Indigenous voices. The use of Yarning was instrumental in this regard, privileging the voices of Aboriginal and Torres Strait Islander Peoples and facilitating a more authentic and culturally sensitive collection of data. By embracing this conversational approach, the study captured a rich, nuanced dataset that reflects the unique experiences and perspectives of the participants. S.B. was a fundamental part of shaping the Yarning methodology and supporting J.C. in her ability to facilitate the Yarning circles. S.B. and J.C. co-facilitated majority of the Yarns. All Yarning circles started with a social Yarn, which then transitioned into research Yarning – incorporating elements of collaborative and therapeutic Yarning. The recordings were transcribed verbatim by J.C. and securely stored.

We consulted with an Indigenous academic colleague (K.W.) who advised us to use a ‘codebook approach’ for our data analysis. Uniquely, we intertwined our codebook approach to also adhere to Braun and Clarke’s 6 steps of Reflexive Thematic Analysis (RTA), as we did not want to disregard the importance of reflexivity within Indigenous research. Therefore, we believe our research approach sits loosely within what Braun and Clarke (2022) call a ‘medium Q framework’ where we have elements of reflexivity as well as elements of structure and rigidity.

Four researchers (S.B., J.C., N.T. and O.W.) underwent data familiarisation (step 1 of RTA), followed by initial coding (step 2 of RTA) of the four same transcripts. We then came together to compare codes and thoughts, then collaboratively generated initial themes (step 3 of RTA). We used a whiteboard to facilitate this task and methodically went through each of the four transcripts and wrote potential codes on a whiteboard. After we went through all four transcripts, we looked at our whiteboard and started forming links between codes and grouped them by a central organising concept. The information from the whiteboard was then used to create a draft codebook (including details of the overarching theme, the codes that sit within each theme, and the definition of each). Next, three researchers (S.B., N.T. and J.C.) were randomly assigned 4-5 transcripts to apply the codebook to independently, using NVivo12 software (as suggested by K.W.). We iteratively updated the codes and themes within the codebook (step 4 using RTA) and recoded as major updates were made. This allowed our codes within our themes to be bound by a central organising concept or shared meaning, rather than topic summaries – which is commonly the case in ‘small q’ and even typical codebook approaches. Once all relevant data was sitting under a code within a theme, we revised theme names and definitions (step 5 of RTA) and then used this to create the basis of the results section of our manuscript (step 6 of RTA). See Supplementary Material (SM) Figure 1 below to summarise what this hybrid approach looked like.


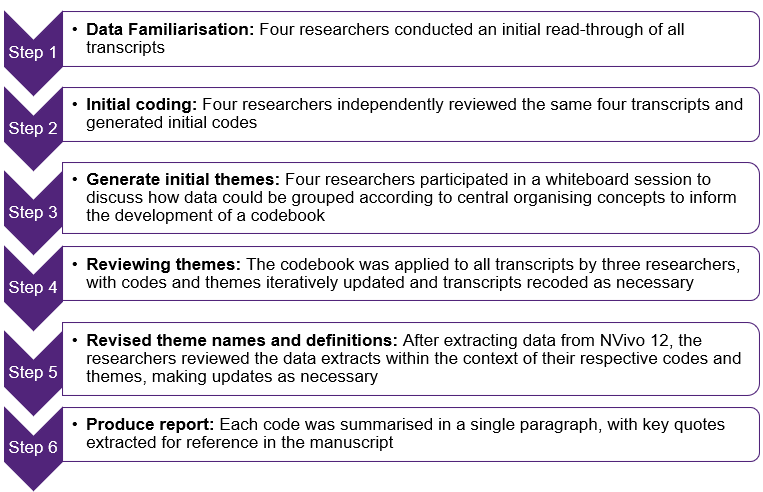


*SM Figure 1: Visual summary of the hybrid codebook-cross-RTA approach used in this research project.*

Throughout this entire data analysis process, we communicated every few days and had at least one meeting each week to discuss any findings or observations and refine or clarify any differences in our coding technique. Having Indigenous representation in these meetings ensured the data was being considered and interpreted in a culturally-sensitive manner by the two non-Indigenous coders. To ensure high quality results and ‘accuracy’, we implemented measures including participant checking (particularly when there were differences between coders in understanding what a participant was saying), consensus coding (loosely – we would just talk through things we were not sure of or wanted a second opinion on), and reflexive journaling. We would bring these reflections to our meetings where we discussed really interesting insights throughout our experience and how it may influence the data. For example, our Indigenous researcher initially found herself slipping between the participants perspective versus offering her own perspective and found it challenging to almost act as an outsider to the participants throughout the analysis process.
