# Supplementary material for: Exploring the perspectives of urban and regional living Aboriginal and Torres Strait Islander Peoples regarding bush foods, nutrition and health: insights for culturally informed health policy in Australia
Source: Public Health Nutr. 2025 Jul 17;28(1):e124. doi: 10.1017/S1368980025100694 (PMC12465064; doi:10.1017/S1368980025100694)
Supplement: Cartwright et al. supplementary material 2 — Cartwright et al. supplementary material [file S1368980025100694sup002.docx]

Codebook

| Theme | Code | Definition and when to use |
| --- | --- | --- |
| Agency v non-agency and links to health behaviour | Ripple effect of self-care and self-worth (or lack thereof) | Use this code for discussions around impacts of having, or not having, self-care. This code is centred around the idea that you always have a choice to break free of a generational cycle of poor health/mindset. This code essentially undermines/opposes the ‘Unhealthy behaviours are normalised’ code. Apply this code when intentional choices are made by caregivers to prioritise health and ensure a healthier lifestyle for children and grandchildren, and/or when intentional choices are made to do the opposite. Use this code when participants discuss ultimately always having a choice/agency to be different/change. |
|  | Education and well-informed health literacy leads to empowerment | Use this code when the participant says a nutrition or health fact or refers to their health/nutrition knowledge. The reason behind this code is to shine a light on the fact this group is well-educated, which may imply the issue is in fact with external barriers not allowing them to act on this knowledge. This code should not be used for Bush Food knowledge – it covers all other aspects of health literacy outside of that. Apply this code not only to participants engaging in dialogue about nutrition and health, but also for participants discussing how this fosters an understanding that enables informed choices. This highlights that education can equip participants with tools to break free from unhealthy patterns, fostering a sense of agency. In other words, there is a transformative power of having knowledge. This code can be used when there is evidence of the importance of education as a catalyst for informed decision-making and empowerment. This may involve any comments alluding to the impacts of health/cooking literacy (e.g. ‘I know soft drinks are high in sugar, therefore I don’t drink it’). |
|  | Rationality v emotionality in food choices and preferences | Use this code for participant comments where we can see the interplay between rationality and emotionality in the decision-making processes. While logical reasoning may inform initial perceptions and judgements, emotional responses ultimately drive behavioural outcomes. Any comments where participants know they are making unhealthy choices can be coded under this. |
|  | Yearning (and willingness) for learning about Bush Foods and nutrition | Apply this code when there is evidence of wanting more education/knowledge on Bush Foods, culture and nutrition. Any comments related to wanting to learn more will be coded as this. |
| Unwavering belief in reciprocity | Intergenerational knowledge transfer among families | To be applied whenever there are discussions around teachings from one generation to another (e.g. parents to kids, kids to parents, Elders to kids, etc.). Not only related to cultural knowledge – any school knowledge or life knowledge should also be included in this code. This code involves discussions and teachings around culture and life – parents, family, or community members wanting to share/pass on this information to someone of a different generation (e.g. teaching things). |
|  | Communal sharing of food and resources | To be applied where there are discussions on inter-community or inter-family sharing of food, equipment, knowledge and culture (e.g. helping fellow Indigenous business start-ups). It is different to the above code for two reasons: 1) use this code where there is lateral sharing of knowledge (e.g. not between-generation), 2) use this code where there is mention of sharing things outside of knowledge (e.g. food, resources, etc.). |
|  | Harmony with nature and symbiosis with the land | Use this code where there is mention of sharing with Country (traditional Indigenous lands) and reciprocity with the land, or discussions around how caring for Country is a mutual exchange where both parties benefit. Use this code for anything to do with treating land (or thinking of land) as an extension of oneself. |
| The socio-environmental grip on food choice | Needing to adjust to a changing food landscape | Use this code for any conversation that mentions the difficulty to maintain a connection with Bush Foods/Country within the confines of modern Indigenous lives. Any comments related to urbanisation or westernisation of the food system can be included here too (e.g. processed foods, being so far removed, etc.), or any comments relating to the shift in food landscape overtime. Use this code for any conversation about the desire for natural (i.e. unprocessed) traditional foods, or for any comments mentioning a change in times (i.e. the ‘new way’ the food system works v the ‘old way’). |
|  | Competing demands means health and culture come second | This code can be applied to data on getting caught up in the demands of life, diminishing the opportunity for cultural interaction and healthy food habits. Any conversations around economic necessities and paying bills taking precedence over cultural teachings or preparing dinner, resulting in a different family dynamic/less healthy choice. Two things can feed into this: 1) incongruency between the way a western capitalist society works v having the time to partake in Country or live a traditional Aboriginal lifestyle, 2) busyness of life impeding on quality of food choices. The idea of ‘labour v return’ applies in this code if in the context of time restraints/convenience. |
|  | The four pillars of food security favour discretionary foods, not Bush Foods. | Use this code for any conversation relating to availability, accessibility, affordability, and stability of different foods. Any discussions about the challenges related to the above in obtaining fresh and nutritious food items, and the enablers related to the four pillars to consumption of unhealthy/sugar-laden choices (e.g. soft drink, spam, bully beef, fried scones, etc.), making Bush Food almost untouchable. |
|  | Unhealthy behaviours are normalised | Use this code for data around the influence of learned behaviours from parents, guardians, friends, or community members as exposure to certain foods such as sugary beverages during childhood can shape perceptions of what is considered normal or acceptable into adult life. Also use this code for conversations around the role of family environments and societal norms in shaping dietary behaviours and attitudes towards food consumption. This code is to do with that unintentional infusion of knowledge and behaviour adoption (which in itself could be a normalised process due to assimilation), rather than intentionally being taught. |
|  | Marketing serves as a powerful tool that shapes our food preferences and choices | Use this code for any comments around how marketing tactics can shape perceptions and drive consumption of certain foods, even without individuals being consciously aware of it. Use this code where the corporate/capitalist influence of dietary choices is discussed – marketing strategies and profit-driven agendas impacting consumer behaviours (particularly regarding the consumption of sugary drinks). Be sure to differentiate between marketing v distrust code appropriately – marketing is about how the thing influences the person (e.g. ‘Coke is red so we can buy it’, Woolworths specials on highly processed foods, etc.), whereas, if a person is saying ‘I don’t eat white bread because it is toxic’ – this is more distrust. The distrust code is less about selling, more about what they are putting in the foods and/or about the behind the scenes and ‘secrets’ companies have and get away with. |
| Bush Foods and Country create opportunities for Mob | Emotional, symbolic and nutritional superiority of Bush Foods | Apply this code when participants talk about the cultural significance and emotional attachment to Bush Foods – highlighting their role beyond mere sustenance. Any comments that indicate food is more than ‘just food’, and comments around feeling ‘proud’ or ‘connected’ when eating Bush Foods and wanting more in their diet. This code shows how Bush Foods can help with emotional, cultural, and spiritual health. Also use this code for conversations regarding the importance of traditional foods in maintaining cultural identity. This code can also be applied to any comments alluding to the high nutritional density or healthfulness of Bush Foods (i.e. how Bush Foods help with physical health). Nutritional superiority is an opinion or fact, whereas the emotion/symbolic/healing code is an effect. |
|  | Bush foods as a viable industry | Use this code for any comments on economic advancements through traditional foods or cultural connections to food (e.g. tour comments, airport comments, supermarket comments, etc.). Also include comments pertaining to the sustainability of Bush Foods, again reinforcing its viability as an industry. Within this code, also include any comments relating to working at the cultural interface to realise this (e.g. ‘the laboratory helps with xxx’). |
|  | Healing power of Country | Use this code for discussions around the impact of being out on Country on individuals’ wellbeing and behaviour. This code suggests that returning to ancestral lands allows individuals to reconnect with their culture, find peace, and escape negative influences. Any conversation discussing the extent to which participants believe this is true should also be included in here (e.g. ‘bush is not a rehab clinic’). Any comments pertaining to how Country is the anchor of everything, Country being described as the classroom for cultural learning, and the importance or outcomes of cultural restoration/healing through Country. |
|  | Traditional Knowledge sharing | Use this code when participants share with the interviewer any information about their culture. For example, Bush Food names, Bush Food knowledge, how to eat/cook/harvest certain Bush Foods, languages, etc. This is different to intergenerational knowledge transfer because intergenerational transfer is discussions around knowledge sharing up/down families and communities, whereas this code should be used when the participant is demonstrating their cultural knowledge to the interviewer. |
| Colonisation erodes culture, and therefore health. | Government decisions lead to poor policy which leads to distrust | This code refers to any barriers to partaking in culture experienced by First Nations Peoples (e.g. red tape, policies, government, etc.), and/or any hurdles First Nations communities face in accessing traditional activities due to restrictions or regulations imposed by government programs or policies. Essentially, use this code to detail mention of any other government interventions or policies. This code highlights the importance of preserving cultural activities as integral components of health and wellbeing. This code should also be used where participants show suspicion or scepticism when the government ‘tries to help’, for any conversations around ‘help’ from the government being not well-received, or for any discussions around the ulterior motive of profit rather than actually helping the community – reinforcing the capitalist western society we live in. Also use this code for conversations on lack of community consultation (or even knowledge) in development of policies leading to unintentional (and sometimes intentional) racism. Finally, this code should also be used where participants talk about wanting transparency over where their food is coming from (e.g. in a western food system, we are much more removed from the food and its processing than traditional First Nations ways of living), or comments relating to how participants perceive the food system. |
|  | Deficit discourse and stereotypes create a negative feedback loop/self-fulfilling prophecy | Apply this code to comments on how challenging it is to escape stereotypes and biases (e.g. if they are treated as if they are less capable, they will be less capable). This code can be used to show that when individuals internalise stereotypes or biases imposed upon them, it leads to feelings of inadequacy (e.g. ‘the colour of your skin is xxx, therefore you must xxx’). This code captures the cyclical nature of stereotype reinforcement, where biased treatment perpetuates the very outcomes it assumes, creating a negative feedback loop that undermines confidence and potential. This code can be applied to conversations around a societal expectation of what being First Nations is, taboo around cultural heritage, experiences of racism, or participants showing a reluctance or discomfort in openly acknowledging and embracing their culture, stemming from historical trauma and/or societal pressures. Also, use this code for any conversations around Mob fighting or disagreeing with Mob. |
|  | Disconnection and separation from cultural identity and Country | This code can be applied in two instances. 1) Use this code for discussions around challenges faced by disconnected First Nations individuals and/or the added disadvantage experienced by those who lack the privilege of cultural teachings. 2) This code can be applied when conversations discuss reasons for disconnection (e.g. systems are ill-equipped to support First Nations individuals, leaving them vulnerable to societal pressures and disconnection from their cultural heritage). Potential topics could be around fractured links to culture, erosion of traditional values, lack of family support, and growing up within systems not designed for First Nations individuals. Use this code for conversations about if participants are on/off Country, living in urban v remote areas, and the impact this has on Bush Food knowledge. |
|  | Intergenerational impacts on health preferences | Use this code for discussions around the historical influence of mission systems on First Nations communities and their dietary practices across generations (e.g. addiction). This code highlights how the ration system has had enduring effects on food consumption patterns within First Nations families. Also use this code for discussions surrounding intergenerational trauma (e.g. family abuse, drug use, etc.), or any discussions evidencing lasting impacts of colonisation that have (whether it be intentional or unintentional) been passed down the family line. Do not use this code for the intentional transfer of intergenerational traditional wisdom – use the code under reciprocity for that. |
