# Supplementary material for: Exploring the perspectives of urban and regional living Aboriginal and Torres Strait Islander Peoples regarding bush foods, nutrition and health: insights for culturally informed health policy in Australia
Source: Public Health Nutr. 2025 Jul 17;28(1):e124. doi: 10.1017/S1368980025100694 (PMC12465064; doi:10.1017/S1368980025100694)
Supplement: Cartwright et al. supplementary material 3 — Cartwright et al. supplementary material [file S1368980025100694sup003.docx]

**Artwork explanation by Sherie Bruce –** **“Bush Foods v Capitalism”**

By creating this artwork, I aimed to represent the fundamental ideas of our collaborative research visually. Each section and symbol were intricately combined to tell a multifaceted story of the intersections between culture, environment, and economy. Central to the design are five black-and-white sections, dividing the canvas into areas that reflect the diverse research themes we explored. These sections are like a mosaic, each contributing to a fuller understanding of our collective study and interlinking different facets of First Nations diets and Traditional Ecological Knowledge.

Encircled within this structured layout are nine dotted circles. Each of these represents one of the nine yarning circles we conducted, where a safe and comfortable environment was created for our participants to share their stories and beliefs freely and openly. The visual narrative is further enriched with four vivid reddish splotches speckled with burgundy dots depicting the fruits and seeds of Kakadu and Davidson plum. These marks symbolise that Bush Food is essential to First Nations diets and the nutrition of our communities.

The artwork incorporates ‘U’ shapes in ochre and orange, representing the gender diversity among our research participants, nine males and eleven females. These shapes represent the active participation of our participants, whose insights and experiences have been crucial in shaping our understanding of the impacts of dietary changes on them, their families, and their communities. At the top and bottom of the composition, circular dots encircled by rings of green and burgundy illustrate my deep connection to my Yolngu and Arrernte communities. These elements underscore the connection to the research topic, anchoring our work in cultural and spatial contexts.

However, this harmony is disrupted by stark black lines that penetrate the artwork from the edges, symbolising the invasive influence of western capitalist food systems. These lines graphically depict the intrusion of colonial and capitalist practices into traditional foodways, illustrating the detrimental effects on First Nations culture and Country.

This artwork is a collective tribute to our research journey. It illustrates the dynamic interplay between Traditional First Nations diets and the pervasive impacts of external economic and colonised forces. It reflects an academic inquiry with a deep commitment to understanding and addressing First Nations communities’ challenges in preserving their cultural heritage and food sovereignty amidst an ever-changing world.

**Artwork Generation**

As an Aboriginal researcher, I have been actively exploring Artificial Intelligence (AI) technologies for the past two years. During this time, I have been an advocate for the removal of Aboriginal art from AI training datasets to protect Indigenous Cultural and Intellectual Property (ICIP). Based on my experience with AI tools, as of September 2024, I have observed that they do not replicate Aboriginal cultural art styles. This informed my decision to utilise AI in creating the artwork, ensuring it does not infringe upon ICIP.

The research team acknowledges the importance of supporting Aboriginal artists and will consider commissioning original artworks in future projects to further uphold and celebrate Indigenous artistry. While commissioning an Aboriginal artist was considered, resource constraints, along with the team’s awareness of my ongoing work in AI-generated art, led us to explore my artistic interpretation of the research. Important to note here is this is something I wanted to do. This approach aligns with my way of thinking, and the final artwork visually represents the exact words I shared with the team during data analysis.

Approximately 50 % of the image was generated by Leonardo AI with the following tool selections: Sampler: Leonardo, Preset: Leonardo Style, Init Strength: No init image, Seed: 5179249342, Fintuned Model: Leonardo Phoenix. Negative Prompts: writing, words. Prompt words: abstract illustration, Australia, bush food, cultural guardianship in the centre, symmetrical, black lines attacking the centre, Aboriginal dot painting styles, intricate patterns and lines, pastel tones, no writing. The generated image was then taken into Canva and Gimp where I (Sherie Bruce) performed further manual digital editing (deletion and addition of elements).

It is important to emphasise that AI was used as a supplementary tool, rather than as a replacement for cultural expression. I have created custom AI models based on my lived experiences and cultural knowledge, with permission from my mother and grandmother (now passed), ensuring cultural integrity and respect.
